# How many surfaces can you distinguish by color? Real environmental lighting increases discriminability of surface colors

**DOI:** 10.1364/OE.531468

**Published:** 2024-09-09

**Authors:** Takuma Morimoto, JoãO M. M. Linhares, Sérgio M. C. Nascimento, Hannah E. Smithson

**Affiliations:** 1 Physics Center of Minho and Porto Universities (CF-UM-UP), R. da Universidade, Braga 4710-057, Portugal; 2 Department of Experimental Psychology, University of Oxford, Anna Watts Building, Radcliffe Observatory Quarter, Woodstock Road, Oxford OX2 6GG, UK

## Abstract

Color supports object identification. However, two objects that differ in color under one light can appear indiscriminable under a second light, a phenomenon known as illuminant metamerism. Past studies evaluated the frequency of illuminant metamerism only under single, uniform illuminants. Here we used computer-graphics techniques to simulate a pair of planar surfaces placed under newly measured hyperspectral illumination maps that quantify the directional variability of real-world lighting environments. We counted the instances of illuminant metamerism that can be solved simply by viewing surfaces tilted to a different direction. Results show that most instances of illuminant metamerism can in theory be resolved for both trichromatic and dichromatic observers, suggesting that the physical directional variability available in natural lighting environments substantially mitigates the biological limitations of trichromacy or dichromacy.

## 1 Introduction

Human perception of surface color is mediated by light reflected from a surface entering the eye and eliciting signals from retinal cones. Importantly, for a single cone, information about the wavelengths of incoming photons is lost in the resultant neural signal (the so-called principle of univariance [1]). This is a fundamental limitation imposed at the very first stage of visual processing, which cannot be undone by any higher-level process. Thus, the sensory input to trichromatic human color vision is limited to triplets of signals from the three classes of cone with different spectral sensitivities [2]. A monochromatic light at 580 nm elicits a yellow percept, but it is possible to generate a color that appears identical by balancing the amounts of two monochromatic lights at, say, 560 nm and 620 nm. In a similar way, the lights reflected from two surfaces that have different spectral reflectances can elicit the same triplet of cone signals under one illumination but a different triplet under a second illumination. This phenomenon, known as *illuminant metamerism*, underlies the difficulty faced by consumers of selecting matching fabric or paint colors in a store only to find that they appear not to match under home lighting. Past studies have shown that illuminant metamerism occurs relatively frequently, which imposes significant constraints on human color vision [3]. For dichromats, who possess only two classes of cones, illuminant metamerism can be an even more severe problem [4].

**Fig. 1:**
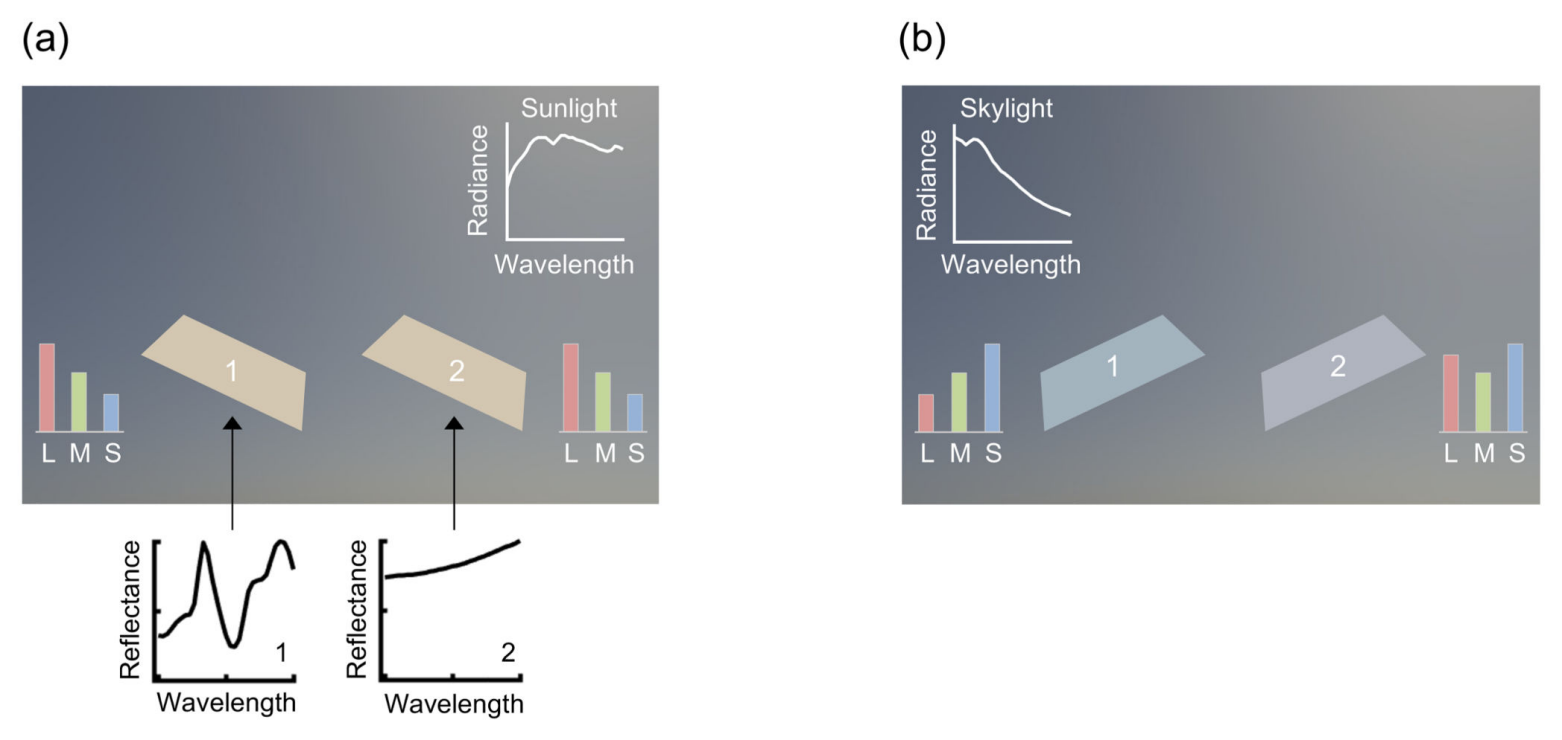
(a) Two surfaces placed under directional lighting environments containing sunlight and skylight are tilted to mainly sample sunlight from the upper right corner in the environment. Their surface colors appear the same to human eyes even though they have distinct surface reflectances. (b) By tilting the surfaces to sample skylight, these two surfaces become distinguishable.

In past studies, however, the prevalence of metamerism has been evaluated under an oversimplified environment where surfaces are uniformly illuminated by a single-spectrum illuminant. Recently collected hyperspectral environmental illumination maps [5] have revealed that natural environments hold significant directional spectral variation; a surface placed in the natural environment samples different illuminant spectra depending on its tilt. This means that a pair of two flat surfaces that appear to have identical surface color at one tilt (Fig. 1 (a)) may be disambiguated simply by adjusting the tilt (Fig. 1 (b)). Here we used computer-graphics techniques to evaluate the frequency of metamerism for planar surfaces placed in a three-dimensional (3-D) environment defined by the measured illumination maps. Furthermore, we evaluated the extent to which natural directional variability increases chromatic discriminability of surfaces.

## 2 Methods

### 2.1 Reflectance datasets

We collected 54,282 reflectances measured from a variety of items: 2,471 flowers, 1,254 fruits, 8,051 human skin samples, 1,498 leaves, and 41,008 man-made objects (e.g., ink or dyes). They were obtained via publicly available datasets [6–8], purchased [9] or via personal communication with Dr. Alexander Shenkin. However, there were many reflectance pairs that were physically very similar. This is problematic because in an extreme case, a pair of two identical surface reflectances is indistinguishable under any illumination, and thus using these spectra leads to an overestimation of the number of metameric pairs. To address this, we reduced the number of reflectances by identifying reflectance pairs that have a Pearson’s correlation coefficient of more than 0.999 in the spectral domain, and removing one reflectance from each such pair. After this procedure 20,132 reflectances (2,143 flowers, 991 fruits, 141 human skin, 1,164 leaves, and 15,693 man-made objects) remained, which were used for the analyses in this study.

### 2.2 Simulation using computer graphics techniques

**Fig. 2:**
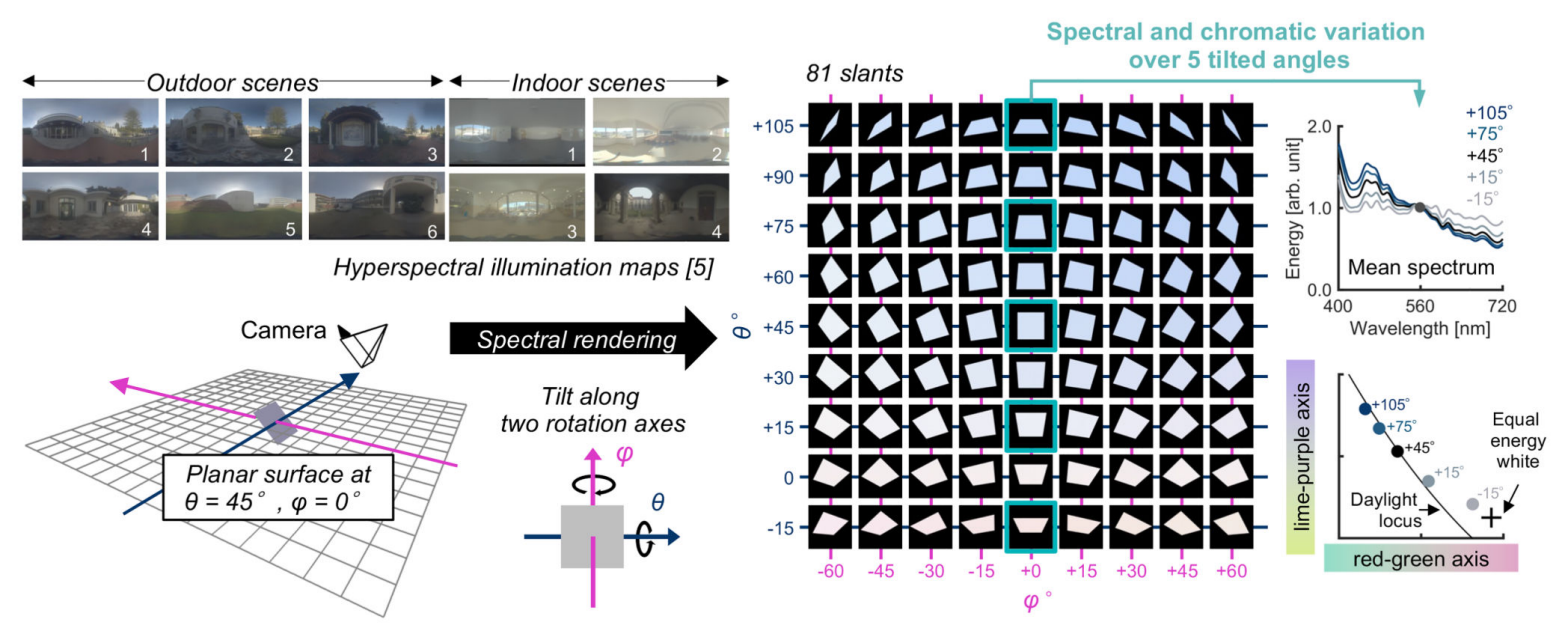
A three-dimensional scene in which a tiltable planar surface was placed. One of ten hyperspectral environmental illumination maps was applied to render the scene. For each of 20,132 surface reflectances, we hyperspectrally rendered the image of the tiltable surface at 81 tilts. The right-hand plots show that the mean spectrum and mean chromaticity change dramatically over the 5 selected tilts.

We used computer-graphics techniques to evaluate the frequency of metamerism. We first constructed a 3-D environment (Fig. 2, left) using the 3-D modeling software Blender (version 2.79, Blender Foundation, Amsterdam, Netherlands). We placed planar surfaces in the scene and applied the measured illumination maps. The surface was matte (no specular reflection) and tiltable so that it could sample spectra from different directions in the scene. We simulated the 20,132 surface reflectances measured and rendered the surface hyperspectrally [10] at 81 tilts (Fig. 2, right) under 10 different environmental illuminations. Depending on the tilt of the surface, the spectrum and chromaticity of the reflected light changes (right-hand plots). The hyperspectral images were converted to XYZ 1931 images, then to CIECAM02-UCS images. We used Brettel’s model to convert XYZ tristimulus values for trichromats to XYZ for dichromats [11]. For each of 2.03 × 10^8^ reflectance pairs (20,132 × 20,131 × 0.5), we computed the Euclidian distance in CIECAM02-UCS space [12], the color difference Δ*E*, at 81 tilts. We then counted the number of surface pairs that were indistinguishable (metameric) at any tilt and calculated the proportion of these that became distinguishable at any other tilt. As a criterion of being distinguishable, we chose Δ*E* = 0.36, which corresponds to the average radius (i.e. just noticeable difference) of MacAdam ellipses in the CIECAM02-UCS space [13]. However, since no official recommendation for the criterion has been provided by the International Commission on Illumination (CIE), we repeated the analysis using different criteria (Δ*E* = 0.5, 1.0 and 2.0) to check the robustness of the result. For each of the 2.03 × 10^8^ surface pairs we considered, we calculated the absolute increase of Δ*E* across 81 slants defined by computing maximum Δ*E* minus minimum Δ*E*. We performed this computation for all lighting environments and concatenated the data separately for outdoor scenes and for indoor scenes.

### 2.3 Perceptual color space

To evaluate whether a pair of surfaces is perceptually discriminable, we need a color space based on human color perception. Past studies have used a variety of metrics to estimate the frequency of metamerism. For example, one early study used Euclidian distance in XYZ tristimulus values [14], while a more recent study [15] chose to evaluate differences in a uniform color space that accounts for the sensitivity of our visual system to color differences in different regions of the space. As mentioned in the previous subsection, we chose to evaluate differences between pairs of colored surfaces in a color space called CIECAM02-UCS [12]. We favored this space over CIECAM02-LCD and CIECAM02-SCD because our objective was to analyze both small and large color differences. There are several parameters that need to be defined to construct this color space. Parameters were set separately for each lighting environment. For the white point *W*_p_, we took the average XYZ1931 values across 81 rendered tilts of a surface that had a uniform reflectance (R = 1.0 for all wavelengths). For the adapting field luminance *L*_A_, we used the mean Y value in XYZ1931 across all pixels in the environmental illumination. For the relative luminance *Y*_b_, we used the ratio between the luminance of the adapting field (*L*_A_) and the luminance of the white point (*W*_p_). These parameters were designed to incorporate the scene’s white point *W*_p_, the luminance level of the background *L*_A_ and observers’ adaptation state *Y*_b_ that are known to modulate color appearance. We set the viewing condition to “average” because this is often used as the condition appropriate for viewing the color of a surface.

## 3 Results and discussion

**Fig. 3:**
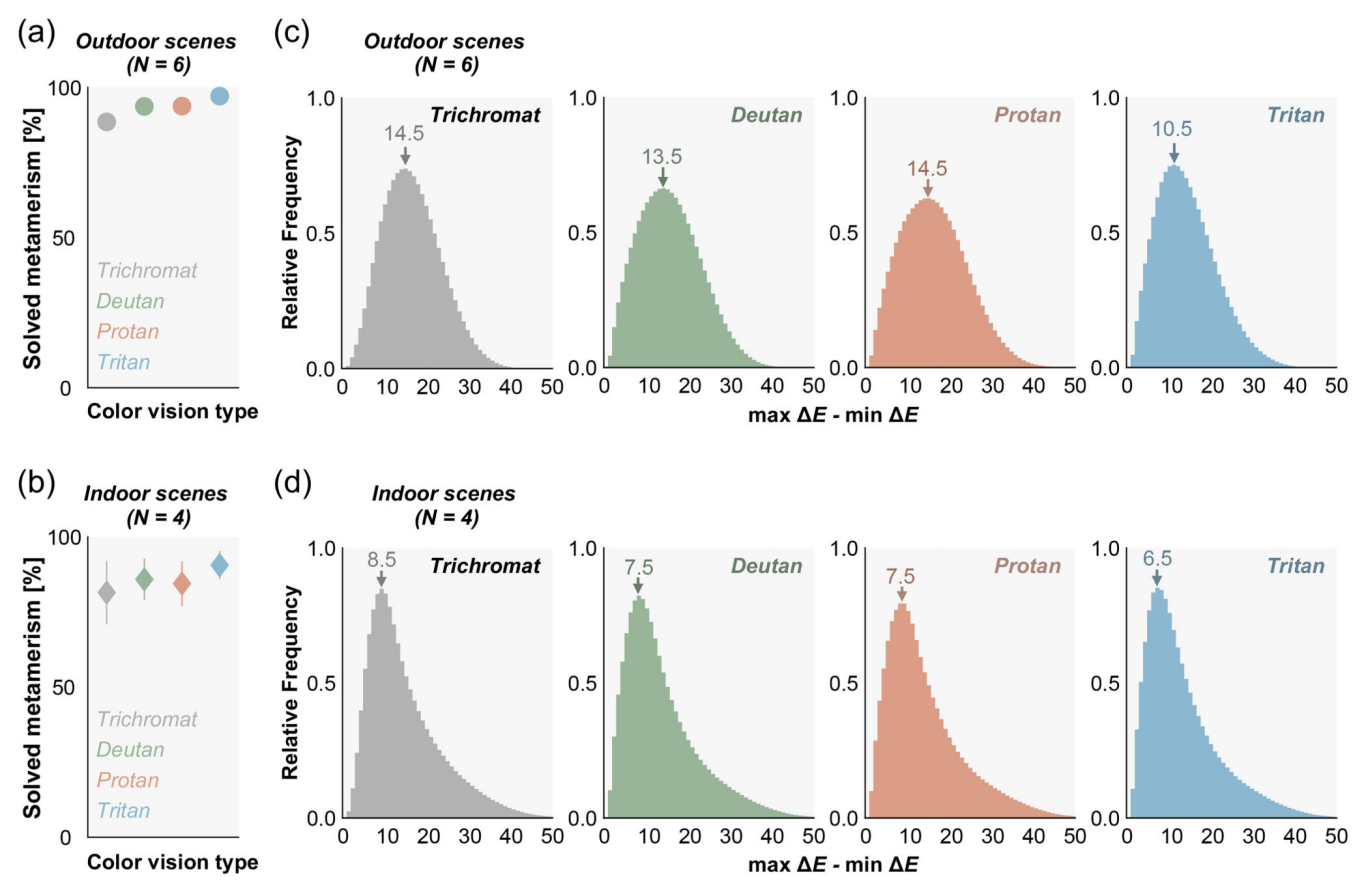
(a),(b) The proportion of metamerism that can be solved by tilting a surface. The color depicts different color vision types. The values were averaged across 6 outdoor environments (a) and 4 indoor environments (b). Error bars show ± 1.0 S.D. across scenes. Δ*E* of 0.36 was used as the threshold for discriminability. (c),(d) Histograms of the absolute increase of Δ*E* defined by the difference between max Δ*E* and min Δ*E*, for outdoor scenes and indoor scenes, respectively. Data were pooled over 6 outdoor scenes or over 4 indoor scenes.

Results showed that trichromats can solve 88.5% of metameric pairs in outdoor environments and 81.5% in indoor environments simply by tilting the surface (Fig. 3, a and b) when the criterion of being distinguishable was 0.36 in Δ*E*. Dichromats can solve an even higher proportion (deutan: 93.6%, protan: 93.7%, tritan: 97.1% in outdoor scenes, and deutan: 85.8%, protan: 84.4%, tritan: 90.6% in indoor scenes). The main effects of scene type (outdoor or indoor) and color vision type (trichromat, deutan, protan or tritan) were significant (*F*(1,8) = 5.40, *η*^2^ = 0.40, *p* = 0.0486; *F*(3, 24) = 18.8, *η*^2^ = 1.54, *p* < 0.00001, where *F*(n,m) represents the F statistic with degrees of freedom n and m in the F-test, *η*^2^ denotes the effect size, and *p* is the p-value) while the interaction between the two factors was not (*F*(3,24) = 0.530, *η*^2^ = 0.0620, *p* = 0.666). Multiple comparisons using Bonferroni’s correction (significance level 0.05) [16] found that the proportion was larger for any type of dichromat than for trichromats, and for tritan compared to deutan and protan. Thus, in real-world lighting environments, illuminant metamerism rarely causes issues for human observers.

Sampling directional spectral variation in real environments yields a more general benefit since it always increases discriminability, regardless of the criterion for distinguishability. Thus, we also evaluated how much Δ*E* between pairs of surfaces increases by tilting the pairs. The mode increase of Δ*E* ranges between 10.5 and 14.5 for outdoor scenes and between 6.5 and 8.5 for indoor scenes (Fig. 3(c) and (d)), confirming that the discriminability of a pair of surfaces dramatically increases both for trichromats and dichromats.

These are the increases of Δ*E* in absolute terms, but the same absolute increase could convey different meanings. For example, an increase from 0.1 to 10.1 and an increase from 10 to 20 represent the same absolute improvement (i.e., plus 10) but relatively speaking the former case shows a more significant change. Thus, we also evaluated the increase in a proportional sense by computing (max Δ*E* - min Δ*E*) / min Δ*E* × 100. We again drew histograms of the metric for each lighting environment and took the mode value, and then computed average mode values across 6 outdoor scenes and 4 indoor scenes. For outdoor scenes, the improvement was 63.5% for trichromats and higher for dichromats (deutan: 99.5%, protan: 85.5%, and tritan: 98.3%). Similarly for indoor scenes, trichromats showed 69.5% improvement and values for dichromats were higher than trichromats (deutan: 89.4%, protan: 86.1%, and tritan: 96.1%). The main effects was not significant for scene type (outdoor or indoor, *F*(1,8) = 0.01, *η*^2^ = 0.0007, *p* = 0.923) but significant for color vision type (*F*(1,8) = 0.01, *η*^2^ = 0.0007, *p* < 0.00001). The interaction between them was not significant (*F*(3,24) = 0.91, *η*^2^ = 0.338, *p* = 0.451). Multiple comparisons using Bonferroni’s correction (significance level 0.05) found that the proportion was larger for any type of dichromat than for trichromats but there was no significant difference among the types of dichromats.

**Fig. 4:**
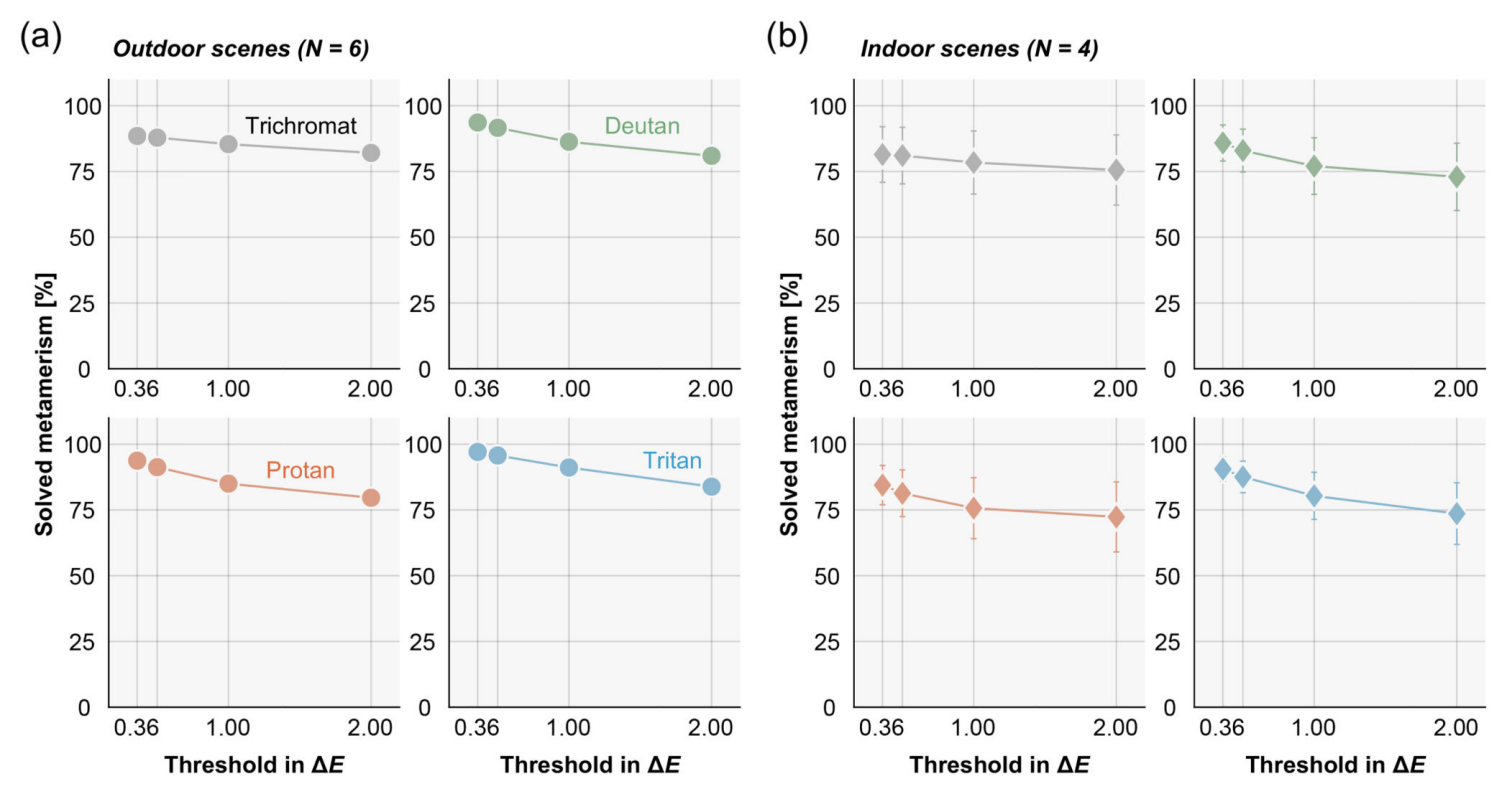
The proportion of metamerism that can be solved by tilting a surface as a function of criterion Δ*E* values at 0.36, 0.50, 1.00 and 2.00. Each panel shows different color vision types. The values were averaged across 6 outdoor environments (a) and 4 indoor environments (b). Error bars show ± 1.0 S.D. across scenes.

Figure 4 (a) and (b) show the percentage of solved metamerism across different thresholds (Δ*E* = 0.36, 0.5, 1.0, and 2.0) for outdoor scenes and for indoor scenes, respectively. We see that the percentages slightly decrease as the Δ*E* values increase for any type of color vision and for both outdoor and indoor scenes. By increasing the criterion Δ*E* from 0.36 to 2.0, the proportions of solved metamerism decrease from 88.5% to 82.1% for trichromats, 93.6% to 80.9% for deutan, 93.7% to 79.6% for protan and from 97.1% to 83.9% for tritan in outdoor scenes. Similarly for indoor scenes, the proportion decreases from 81.5% to 75.6% for trichromat, 85.8% to 73.0% for deutan, 84.4% to 72.4% for protan, and from 90.6% to 73.6% for tritan. However, importantly the general conclusion that a large proportion of metamerism can be solved still holds.

Illuminant metamerism arises from univariant cone responses. In this sense, it has been taken as a hard limit on human color vision. In addition to its biological significance, the metamerism problem has practical implications in industries such as printing and textile manufacturing, where color control under different lighting conditions is crucial. However, we show that directional spectral variation in natural environments allows us to distinguish most of the reflectance pairs that are indiscriminable under a single-illuminant. These results add a new perspective to classic trichromatic theory [2] that serves as a basis of modern color vision research. The term trichromacy may imply that we have access to only three sensory inputs to create color, but this is true only when we consider a snapshot of our external world. Instead, in daily lives cone signals associated with a particular surface change in a dynamic way. Even if we are missing one type of cone, we might recover the ‘missing’ dimension by using signals sampled at different time points [17].

Statistical tests suggested that the directional variation in the spectral content of natural lighting might provide greater benefits to dichromats than to trichromats. To explore the reasons behind this observation, we additionally performed analyses within individual categories such as flowers, fruits, human skin, leaves, and man-made objects. The results revealed that trichromats exhibited a higher rate of solving metamerism for natural objects (skin, fruits, and leaves), while dichromats showed a greater advantage for man-made objects. The higher proportion of man-made objects in our dataset may therefore have contributed to the overall higher solving rate observed for dichromats. It should also be noted that the types of objects included in the natural and man-made categories could influence the results. For instance, a dataset rich in objects aligned with the deutan confusion line under one of the sampled illuminants might show a greater benefit of tilting surfaces for deutan observers. Thus, the comparison of trichromats and dichromats based on the present dataset should not be over generalized. Despite the differences indicated by the ANOVA results, the most important finding is that both trichromats and dichromats were capable of solving a substantial proportion of metamerism cases and of increasing discriminability of surface colours by tilting surfaces in natural lighting environments.

There are several studies that have examined the number of perceptually distinct surface colors [18,19], and one might wonder about the difference between these studies and those on illuminant metamerism. First of all, studies on the number of distinguishable colors generally do not consider the influence of illumination. Instead, they focus on counting the number of distinguishable colors under a fixed illuminant condition, considering the limits of human color discrimination. In contrast, research on metamerism centers around the influence of illumination, examining how external factors such as illuminant change interact with the limits of the human color vision system. Regardless of whether the focus is on the number of discernible colors or on metamerism, past studies have not considered the possibility of actively sampling the different illuminants available in natural lighting environments. Our study goes further by considering how dynamically available signals can mitigate the problem of illuminant metamerism.

Precise measurements of directional spectral variation of the illumination in natural environments allowed us to perform the present analysis for the first time. Moving forward, it will be important to empirically test whether the visual system can use directional light to support surface color discrimination. However, the use of a perceptual color space in the modelling indicates that the magnitude of the physical effects do indeed translate to perceptually meaningful differences that could support discrimination performance. Other limitations include the use of an ideal model surface that does not capture all the complexity of natural objects. Moreover, the present study did not incorporate the complex sampling behavior of human observers interacting with objects when discriminating surface colors. For example, dichromats might be motivated to explore surfaces in a different way from trichromatic observers, to improve their discrimination. Even considering these limitations, we show through simulation that our visual system can benefit from the additional information gained by simply tilting the surfaces.

Some recent studies have suggested that illuminant metamerism is relatively infrequent when natural reflectances and illuminants are considered [15,20]. In fact, when we counted the number of pairs where Δ*E* was lower than 0.36 at any tilt, we found that the proportion of such pairs relative to the total number pairs (2.03 × 10^8^) was 6.23× 10^−5^ for trichromats, 8.88× 10^−4^ for deutan, 8.52× 10^−4^ for protan, and 1.31× 10^−3^ for tritan (i.e. 1.27 × 10^4^, 1.80 × 10^5^, 1.73 × 10^5^ and 2.66 × 10^5^ pairs, respectively). Here we find that metamerism is even less frequent because of directional spectral variation in real world lighting. Raising this as a theoretical possibility is not new [17], but what is new is the formal quantification of the extent of this possibility in real lighting environments, made possible using new light-measurement data and computer-graphics rendering techniques that allow spectral simulation of light-material interactions. It is striking that the complexity inherent in natural lighting environments can compensate for the biological limitations of trichromatic and dichromatic visual systems to a large extent. In addition to the scientific importance, we make the newly measured hyperspectral images of indoor and outdoor scenes freely available. We anticipate that this dataset will be of interest to many researchers in diverse fields of ecological optics and vision science, who are interested in the statistical characteristics of natural lighting environments. Furthermore, in the commercial sector, such as the paint and textile industries, data that captures illuminant directionality will help development of materials that behave in desired ways when embedded in such lighting environments.

## 4 Conclusion

Illuminant metamerism restricts the number of surface colors we can distinguish. Using a newly measured dataset that quantifies the directional variability of real-world lighting environments, we analyzed how much illuminant metamerism could be resolved simply by tilting the surface. Results demonstrated that most instances of illuminant metamerism can be resolved for both color-normal and color-deficient observers. Furthermore, regardless of the threshold criterion for distinguishability, the discriminability of a given surface pair can significantly increase. These findings provide new insights into how the color vision system benefits from the complexity inherent in natural lighting environments.

## Data Availability

Hyperspectral environmental illuminations are available at [21].
